# Revealing the ancient origins of blonde beers: Phylogeography and phylogenetics of cryotolerant fermentative yeast *Saccharomyces eubayanus* from pre-Hispanic pottery in Northwestern Patagonia, Argentina

**DOI:** 10.1371/journal.pone.0319938

**Published:** 2025-04-11

**Authors:** Alberto E Pérez, Jacobo Hernandez-Montelongo, Melisa González Flores, María Eugenia Rodríguez, Christian A Lopes, José Luis Lanata

**Affiliations:** 1 Facultad de Ciencias Sociales y Humanidades, Universidad Autónoma de Chile, Temuco, La Araucanía, Chile; 2 Núcleo de Investigación en Bioproductos y Materiales Avanzados, Departamento de Ciencias Matemáticas y Físicas, Universidad Católica de Temuco, Temuco, La Araucanía, Chile; 3 PROBIEN, CONICET-UNCo, Neuquen, Argentina; 4 IIDyPCa, CONICET-UNRN, Bariloche, Río Negro, Argentina; University of Johannesburg, SOUTH AFRICA

## Abstract

This study presents an analysis of residuals and the identification of the oldest cryotolerant fermentative yeast Saccharomyces eubayanus, absorbed in the walls of ceramic vessels. The samples were dated between 920 and 750 years before present (BP) from the Meliquina Lake site in northwest Argentine Patagonia. This study provides more solid evidence supporting the hypothesis of a pre-Hispanic development area for fermented beverage production at the southernmost region between $-38^{\circ }$ and $-40^{\circ }$ south latitude on the continent. The isolation and subsequent phylogenetic and phylogeographic analysis of this yeast strain confirm its primitive nature, predating the previously known European hybrids. The associated context and chronology of its use, predating European-Indigenous contact, provide evidence of its management and utilization in native or autochthonous fermentative processes. Subsequently, for reasons still unclear, the strain migrated to Europe, where it hybridized with Old World strains, culminating in the emergence of blonde beers or lager in 16^*th*^ century Bavaria. The deliberate or unintentional nature of this migration remains speculative, but it underscores the significant role this yeast strain played in the development of one of today’s most popular fermented beverages, which necessitate fermentation at low temperatures.

## Introduction

The production of fermented alcoholic beverages has played an essential role in the history of our species during the Holocene period, serving as a significant source of nutrition, drinking water, medication, and practices that stimulated the consolidation of intra- and intergroup social relationships through the act and ritual of drinking [1,2]. Simultaneously, it was intertwined with emerging technologies and various economic and social practices such as pottery, trade, the promotion of individuals and lineages, and the adoption of sedentary lifestyles [3–5].

### Concerning the evidence of fermented beverages

The archaeological evidence of the production and consumption of alcoholic beverages is complex. While it is an integral part of a society’s material culture, involving the management and transformation of natural resources and the transmission of knowledge about the structure of plant resources [3], alcoholic beverages are perishable overtime [4]. As such, the identification of these beverages in archaeological contexts is challenging [4], not only due to the evaporation of alcohol but also because the non-volatile residues (both organic and inorganic) undergo alterations during the processing stages, leading to limited survival of macro (carpological) and microfossil evidence (such as silico-phytolithic, pollen), among others [4,6,7]. However, when these residues preserved in pottery and dental calculus [4], characteristic fermentation attributes can be sought [4,6], in both the residues and the containers themselves. Attributes such as thread enlargement, damage to the edges, gelatinization, fissures, perforations, birefringence, fractures, and rough texture of the pollen, for example, can be used to postulate fermentation [6,7]. Nevertheless, these attributes are general derivatives of exposure to heat, roasting, dehydration, and grinding of grains and seeds, and thus can also result from food preparation processes, not exclusively linked to the production of fermented beverages, unless supported by other evidence. For this reason, it becomes necessary to identify known alteration patterns in potential containers, such as chipping on the edges and detachment of the interior surfaces of ceramic vessels, resulting from erosion produced by acidification during the fermentation process [4,8]. These patterns may also be associated with treatments to improve the alkalinity of maize, for example, used in the production of fermented beverages [9].

Fermented beverages represent a distinct type of material culture [3] linked to the development of other materialities, such as the diversity of pottery technology [10]. Vessels serve as potential containers for grain, legume, and fruit ferments, enabling storage and preservation for deferred consumption, especially for a wide range of foods that are abundant but only seasonally available [1,11,12]. As Hayden [1] postulates, technological innovations such as ceramics are closely related to feasts in resource-rich areas that exploit abundant resources and are less vulnerable to overexploitation [10].

### Exploring the Southernmost production of fermented beverages of the Americancontinent

Since 1550, historical sources from the region [13–15] have described the practice of annual meetings and/or gatherings among the Reche-Mapuche populations in the south-central region of Chile. These events were translated by Hispanics and Dutch conquerors as “Indian boards,” “leagues” and “congregations,” directly alluding to the significant consumption of alcoholic beverages, often referred to as ‘bebederos.’ These ceremonial feasts could last between 15 and 20 days, involving a large number of people and goods manufactured in ceramics, frequently serving as containers for service fermented drinks and food [16].

Furthermore, ethnographic sources from the early $20^{th}$ century mention the native production of fermentative drinks among the Mapuche population, considered an ancestral practice of great ritual value [17]. These sources describe the production sequence, including grinding, boiling, and fermentation of fruits and grains in ceramic vessels [18,19]. The documentary sources also mention that women primarily undertook the preparation of these beverages, often in the context of domestic production. However, their service typically occurred during collective public events, such as ceremonial feasts [5,6].

### Archaeological sites in the North Patagonian Andean temperate forests

#### Western Andean sector (Chile).

**Fig 1:**
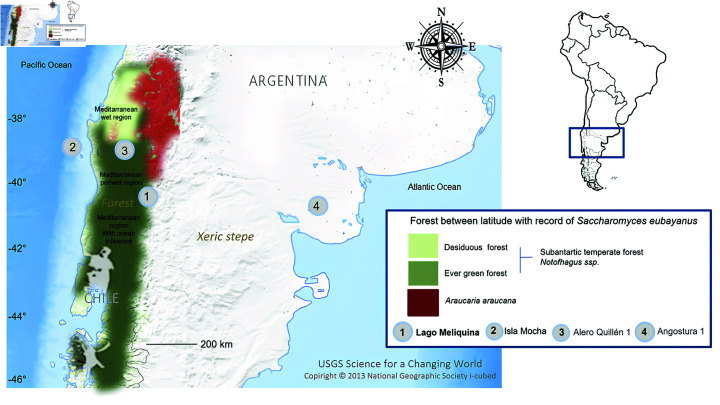
Geographic distribution of *Saccharomyces eubayanus* records and archaeological sites with evidence of fermentation vessels: (1) Lake Meliquina, (2) Isla Mocha, (3) Quillén 1, and (4) Angostura 1. Image was builded from USGS database.

Until now, the possible production of fermented beverages at $-38^{\circ }$ South in the western Andean sector has been just suggested [6]. This hypothesis was based on the identification of attributes or superficial and structural modifications of microfossil residues of pollen from wild and domesticated plants (including corn) found in El Vergel ceramics dating between 1,000 and 1,300 AD, from sites in the insular contexts of Mocha Island (Fig 1). However, the results of the microfossil analysis were previously solely compared with morphological/functional typological characteristics of containers such as vessels, pots, jars, etcetera [6]. These analyses have not yet incorporated attributes or modifications on the surfaces associated with fermentation, as described in the archaeological literature [4]. To be more precise, maltose residues were recently identified in the walls of El Vergel ceramics at the Quillén 1 site, located in the middle Cautín river basin at $-38^{\circ }$ south, within the central valleys of the continental sector (Fig 1).

#### Eastern Andean sector (Argentina).

Based on the initial archaeological works conducted at the Lago Meliquina site [10,20], it was postulated that the quantity and diversity of morphological groups of ceramics at this site align with the expectations for complex hunter-gatherer societies that employ pottery in rituals or feasts [1,21]. These societies typically leave behind a pottery record consisting of vessels not only intended for containment and food processing (pots) but also used for serving food and beverages, such as pitchers, bowls, glasses, bottles, plates, among others [5,10], during ceremonies similar to those previously described in historical sources from the region. In our study area, the ceramic samples are primarily characterized by utilitarian wares, such as medium-sized pots, pitchers, and cups with polished surfaces. These ceramics are sparsely decorated, featuring black-on-red reserve painting, linear designs, and, to a lesser extent, punctate incisions. The forms are predominantly closed, meaning the mouths are smaller than the largest diameter of the bodies [22], with polished external surfaces and smoothed interiors. This context reflects a subsistence strategy still dominated by hunting and gathering, complemented by food production, which remains under-researched and minimally represented [23]. In this setting, the potential fermentation of fruits and seeds may have been an efficient way to utilize abundant but seasonally available plant resources [23]. Such practices likely emerged within complex hunter-gatherer societies that began to store and process resources for delayed consumption or special occasions, possibly serving as prestige goods for ceremonial use or for the promotion of social status.

Recently, evidence of alterations in silico-phytolithic residues and internal rims of monochrome vessels associated with the containment of fermented beverages has emerged from lower archaeological levels dated 938 ± 45 BP at the Angostura 1 site (Fig 1), located in the upper basin of the Negro River at $-40^{\circ }$ south [7]. These findings pertain to pottery hunter-gatherer societies, suggesting the potential fermentation of locally available carob (Neltuma sp.) seeds and pehuén (*Araucaria araucana*) pine nuts from the Andean Cordillera sector (our study area).

### *Saccharomyces eubayanus* in Patagonia

Yeasts belonging to the genus *Saccharomyces*, particularly the species *Saccharomyces cerevisiae*, are associated with various food processes worldwide, including baking, distillation, and the production of wines, beers, ciders, and other fermented foods [24]. In the case of *Saccharomyces cerevisiae*, in Patagonia, is currently linked to wine and cider production environments [25]. Another species of the genus, such as *Saccharomyces uvarum*, is also involved in fermentative processes and has been isolated from natural habitats in Patagonia [2,26] as well as apple chichas [26] and ciders fermented at low temperatures [27] in the region.

The classification and analysis of fermentative yeasts involved in the production processes of some of the most commercialized alcoholic beverages, such as lager beer, have sparked interest in investigating the selective processes worldwide [2,26,28]. In 2011, an interdisciplinary team discovered a previously unknown cryotolerant yeast species at $-37^{\circ }$ south latitude in Patagonia, Argentina (Fig 1) in Nothofagus forests (*Nothofagus pumillo*, *Nothofagus antartica*) and fungus *Cyttaria sp* parasitizing these Nothopagus species. Previous studies had recognized that, when this combined with *Saccharomyces cerevisiae*, this yeast species gave rise to the hybrid yeast allotetraploid *Saccharomyces pastorianus*, a species that does not occur naturally and is directly involved in the production process of lager beer [2]. This new species was named *S. eubayans* [2].

Subsquently, Rodríguez et al. 2014 extended the natural distribution of *Saccharomyces eubayanus* to parallel $-40^{\circ }$ south latitude and to other host species, specifically *Araucaria araucana*. More recently, Chilean researchers, alerted by these discoveries, successfully searched for and identified the yeast in the western Andean sector, specifically in the south-central area of Chile [28]. Moreover, new fieldworks led to the establishment that two previously registered cryotolerant species, *S. eubayanus* and *S. uvarum*, seemingly coexist in sympatry in southern Nothofagus forests. However, these species are genetically isolated due to intrinsic and ecologically potzygotic barriers, and determined by host preference [2]. Nevertheless, this data is partially refuted by the subsequent record of these species in the bark and pinion of *Araucaria araucana* [26]. This discovery raised the hypothesis of their involvement in the fermentative process of traditional native beverages based on pine nuts, known as “Mudai” [17]. However, the results of the respective investigation showed that currently, their production among the Mapuche indigenous peoples is exclusively associated with commercial species such as *S. cerevisiae*, which is also used in domestic and commercial bakery at the regional level [26].

On the other hand, in a recent study conducted by our group, we successfully isolated and characterized various strains and genomic populations of the species *S. eubayanus* [29]. These strains were discovered in fermentative environments closely associated with apples; a fruit introduced to America by the Spanish in the $16^{th}$ century. Ethnohistorical records suggest that since the $18^{th}$ century, fermentation of apples became popular among the Mapuche populations, gradually replacing other native fruits and seeds in preference [17]. This work included isolation sites such as apple chichas, an ancestral beverage produced by indigenous communities, as well as feral apple trees, both located in Aluminé city (Neuquén, Argentina). This groundbreaking discovery marked the first isolation of the species in a fermentative environment [30].

In that sense, this study aims to connect the ancient origins of blonde beers by investigating the phylogeography and phylogenetics of the cryotolerant fermentative yeast *Saccharomyces eubayanus *from pre-Hispanic pottery in Northwestern Patagonia, Argentina. For that, we identify evidence of pottery used for processing, containing, and/or serving fermented beverages, shedding light on the diversity of ceramic containers in the early pottery history of the region. It also provides empirical insights into indigenous fermentative beverage production processes. These processes may have contributed to the transmission of cryotolerant fermentative yeasts to Europe through interactions between indigenous populations and European conquerors, potentially facilitating hybridization. Such interactions, whether intentional or not, could have played a role in the development of beverages fermented at lower temperatures, such as blonde or lager beers.

The consumption of fermented beverages in the extreme south of the American continent has been inferred indirectly, suggested by the presence of abundant service ceramics and alterations in the internal surfaces of ceramic vessels. Additionally, adhered substances, such as pollen microremains, offer further clues. The ceramics, including pots, pitchers, and cups, found in the study area, are likely associated with the production, containment, and/or serving of fermented beverages. This could be evidenced by the presence of specific indicators, such as cryotolerant yeasts linked to low-temperature fermentation processes. Further support may come from residues showing alterations, such as changes in pollen and phytoliths, which are also linked to these fermentation practices.

## Materials and methods

### The sample

The samples were collected from surface archaeological sites and forested stratigraphic contexts containing ceramic remains, including Lake Meliquina, Cave Parque Diana, Los Radales 1, Catritre, Siete Manzanos, and Newen Antug (Fig 1). These sites, where native fermentative yeasts were recently identified [31], were analyzed to evaluate the preservation of organic material. The research employed a two-pronged approach: first, exploratory analyses were performed in forested sites with abundant pottery to test the conservation of yeasts using reagents; second, samples were carefully selected based on their archaeological context and preservation state, enabling more detailed investigations, including genetic analyses. The artifacts were collected using a stratigraphic intervention method involving grids measuring 1 × 1 m, and the excavation reached a sterile level of archaeological artifacts between depths of 0.10 and 0.30 m and 0.60 and 0.70 m. Exactly 100 samples of different layers were extracted of a total of more than a thousand ceramic artifacts in the sites, protected to preserve potential organic residues, such as fatty acids and DNA. These samples were analyzed at the microbiology laboratory of PROBIEN, CONICET-UNCo. Samples were recovered and separated in sterile conditions in the field, protected, unaltered (not washed or labeled) and stored under controlled light, temperature, and humidity conditions to avoid compromising the integrity of any organic residues for future analysis, including fatty acids, stable isotopes, genetic/molecular, radiocarbon, toxicological, among others.

All samples consist of fragments from containers classified as pots and jars, including sections of body segments, bases, and rims. A subset of 43 fragments was selected for detailed analysis (Table 1). Perimeter cuts were made under sterile conditions and biomolecular laboratory protocols to reduce the samples to 1 cm^2^ fragments. This process involved discarding edge sectors, which are more exposed to natural contamination factors during both stratigraphy and archaeological recovery. Subsequently, the samples underwent surface disinfection through exposure to UV light for 5 min. Control samples were collected from surfaces in direct contact with sediments, then the ceramic surface was removed to a depth of 2 mm using a scalpel.

**Table 1:** Identified yeasts in the archaeological ceramics: *Aureobasidium pullulans* (Ap), *Filobasidium oeirensis* (Fo), *Holltermaniella wattica* (Hw),* Naganishia antarctica *(Na), *Naganishia bhutanensis* (Nb),* Naganishia cerealis* (Nc), *Naganishia diﬄuens* (Nd), * Saccharomyces eubayanus* (Se), *Solicoccozyma aerius* (Sa), *Solicoccozyma phenolicus* (Sp), *Vishniacozyma tephrensis* (Vt).

Yeast species														
No.	Site	Coordinates	Chronology	Ap	Fo	Hw	Na	Nb	Nc	Nd	Se	Sa	Sp	Vt
1–3	Mirador de Bello	40°09’13”S 71º17’28”W	Historical	—	—	—	—	—	—	—	x	x	x	x
4–6	Catritre	40°10’29”S 71°23’43”W	Historical	x	—	—	x	x	—	x	—	—	—	x
7–9	Siete Manzanos	40°08’14”S 71°13’45”W	850 ± 60 BP	—	—	x	—	x	—	—	—	—	x	—
10–12	Fuente de Arcillas	40°01’50”S 71°22’42”W	Historical	x	—	—	—	x	—	—	—	—	—	—
13–15	El Nido 2	40°04’42”S 71°19’21”W	Historical	—	—	—	—	—	—	—	—	x	x	—
16–18	Los Radales 1	40°09’32”S 71°18’44”W	480 ± 60 BP	—	—	—	x	x	—	—	—	x	x	—
19–21	Quechuquina 3	40°10’02”S 71°34’39”W	Historical	—	—	—	—	—	—	—	—	—	—	—
	Cave Parque Diana													
22–24	CPD upper level	40°19’00”S 71°22’28”W	580 ± 60 BP	—	—	—	x	—	—	—	—	—	—	—
22–24	CPD upper level	40°19’00”S 71°22’28”W	760 ± 60 BP	x	—	—	—	x	—	—	—	—	—	—
25	CPD middle level	40°19’00”S 71°22’28”W	900 ± 60 BP	—	—	—	—	—	—	—	—	—	—	—
25	CPD middle level	40°19’00”S 71°22’28”W	990 ± 60 BP	—	—	—	—	—	—	—	—	—	—	—
	Newen Antug													
26–28	NA (AII, P5z)	40°09’44”S 71°20’49”W	540 ± 50 BP	x	x	—	—	—	—	—	—	x	—	x
29–31	NA (sondeo N1)	40°09’44”S 71°20’49”W	880 ± 40 BP	—	—	—	—	—	x	—	—	—	—	—
	Lake Meliquina													
37–39	LM-FM, S2 (BI, P4)	40°20’03”S 71°19’08”W	920 ± 60 BP	—	—	—	—	—	—	—	x	—	—	—
40–41	LM-FM, S2 (BI, P6)	40°20’03”S 71°19’08”W	730 ± 60 BP	—	—	—	—	—	—	—	x	—	—	—
42–43	LM-FM, S1 (BIV, P2)	40°20’03”S 71°19’08”W	730 ± 80 BP	—	—	—	—	—	—	—	x	—	—	—
42–43	LM-FM, S1 (BIV, P3)	40°20’03”S 71°19’08”W	750 ± 60 BP	—	—	—	—	—	—	—	x	—	—	—

The analyzed artifacts are housed in the repository of the Municipality of San Martín de los Andes (Gral. Roca and Juan Manuel de Rosas -8370- San Martín de los Andes, Neuquén Province, Argentina) under registration numbers LAE-NQ-NA, Alf: 009/51 (Ceramics). Research in the region began between 2008 and 2012 within the framework of projects conducted by the National Parks Administration (DRP-APN Nº834) and the University of Buenos Aires (PRI-UBA 840162), focusing on the archaeology of the southern Neuquén forest and its connections with sites in the Paso Limay area, the Río Negro steppe, and transitional sectors. From 2012 to 2015, the work was carried out by archaeologists and technical staff from the Laboratory of Archaeology and Ethnohistory (LAE) under the Secretariat of Planning and Sustainable Development of the Municipality of San Martín de los Andes (Neuquén Province, Argentina). The artifacts are stored in the annex repository of the municipal facility for registering and conserving heritage assets, in compliance with Article 24 of Law 2184 on the Historical, Archaeological, and Paleontological Heritage of the Province of Neuquén. Additionally, sacred objects of the local Mapuche community are protected under Law 25.517. As the local enforcement agency, all necessary permits for the study of the ceramic materials were obtained through Municipal Decree 1131/12, ensuring full compliance with applicable regulations.

The pottery associated with the studied sites and the Pitren tradition, in general, is well-fired, though not necessarily at high temperatures. As a result, the pottery tends to be relatively soft and of low hardness. Typically, the external surfaces are polished, while most of the closed forms feature irregularly smoothed and porous internal surfaces. This characteristic allows obtaining powdered ceramic material was extracted from the interior of the ceramic cores by friction using another scalpel blade. This material was placed in sterile tubes containing glucose-yeast-peptone (GPY) culture medium (% w/v: 2 glucose, 0.5 peptone, 0.5 yeast extract)-sorbitol 1 M with chloramphenicol (100 mg/L). The tubes were incubated at $20^{\circ }$C under shaking. Microbial development (turbidity) was observed in some of the tubes, confirming the presence of yeasts through microscopic observation.

To identify the presence of fermentative beverages, we aimed to detect specific attributes, such as the presence of native yeasts associated with fermentation in cold environments (cryotolerant). These yeasts can be directly linked to the production, storage, or serving of fermentative beverages. In that sense, to perform taxonomic analysis, aliquots of yeasts suitable dilutions (0.1 mL) were spread onto GPY agar (g/L: 0.05 yeast extract; 0.05 peptone; 0.2 glucose; 0.2agar-agar) supplemented with chloramphenicol (200 mg/L). After incubation for 2–5 days, colonies were picked and stored in a glycerol 20% v/v solution at $-20^{\circ }$C. Colonies were taxonomically classified on the basis of the sequences obtained for the D1/D2 domain in the 26S rDNA region following the methodology proposed by [32]. PCR products were submitted to a sequencing service (Macrogen Korea) and subsequently compared using BLASTn with sequences of the type strains available in the NCBI database.

### Phylogeographical and phylogenetic analysis

The analysis of the *S. eubayanus* strain isolated from Lake Meliquina was carried out with the *intFR *partial sequences. PCR product was cleaned using the AccuPrep PCR purification kit (Bioneer, Inc, USA) and subsequently submitted to a sequencing service (Macrogen Korea) under accession numbers PP31932 (Fig 4a, https://www.ncbi.nlm.nih.gov/nuccore/PP319392). The access data for all the genetic samples are available in the his region was previously described as highly variable intergenic region [33] and has been utilized to characterize different subpopulation relationships by [29]. A total of 71 strains isolated from Patagonia (Chile and Argentine) in both natural [28,29] and fermentative [30] environment were included to the analysis. The complete set of homologous sequences was aligned with the ClustalW program [34].

### Neighbor-Joining (NJ) analysis

The best evolutionary models were selected using JModelTest 2.1.10 [35], and the Tajima-Nei model (1984) [36] was identified as the best-fitting model for our dataset. The Tajima-Nei model is particularly appropriate as it accounts for differences in nucleotide substitutions and base pair frequencies, which aligns well with the moderate sequence divergence observed in yeast populations. Phylogenetic trees were reconstructed using the Neighbor-Joining (NJ) method [37] with 1000 bootstrap replicates for branch support, performed in MEGA7 [38]. This methodological approach effectively highlights the spatial and temporal relationships of the yeast..

### Median-Joining network (MJ)

Haplotype classification was conducted using DnaSP v5 [39]. Median-joining (MJ) networks were obtained using Network 4.5 [40].

## Results

Our results demonstrated the preservation of yeasts in this type of pottery samples recovered from these forested archaeological sites. Consequently, yeast strains were isolated using GPY-agar plates and identified through molecular techniques, including PCR-RFLP and sequencing. The analysis revealed the presence of 11 yeast species (Table 1), including the species *Saccharomyces eubayanus*, which was isolated in two sites, namely, Mirador de Bello and Lago Meliquina (Fig 1). Subsequently, intraspecific characterization of the isolates was performed using mtDNA-RFLP [25]. The results indicated the presence of a majority strain (with identical molecular profiles) in isolates obtained from both sites. One colony, isolated from Lago Meliquina, was selected for phylogeographic and phylogenetic analysis.

### Sites with records of *Saccharomyces eubayanus*

Mirador de Bello is a single-component site with multiple open-air activities, situated on the north slope of the Chapelco range, near the Maipú Valley. This newly discovered site is currently in its initial stages of study and exhibits lithic artifacts, including stalked projectile points, and utilitarian pottery typical of the pre-Hispanic Late Pottery Period (1,000 to 1,550 AD) [5]. The second site, Lago Meliquina, is a unicomponent open-air settlement dated between 92060 BP, 750 ± 60 BP, 730 ± 50 years AP [20]. Since the sample from Lago Meliquina was the sole specimen acquired through stratigraphic excavation, our primary focus was on this sample. Additionally, we provided descriptions of other organic and inorganic evidence that could have been linked to the production of fermented beverages and the potential utilization of *Sacharomyces eubayanus*, as these pieces of evidence originated from the same ceramic sample.

### The context associated to Lake Meliquina samples

**Fig 2:**
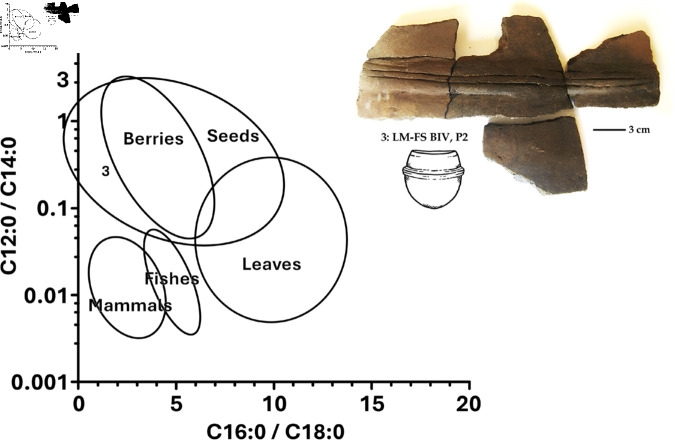
Seed/berry identification among pottery fatty acids from the Lago Meliquina site [31] testing positive for *S. eubayanus.* Sample No. 3: LM-FM-S1, BIV, P2 corresponds to No. 42 and 43 in Table 1.

**Fig 3:**
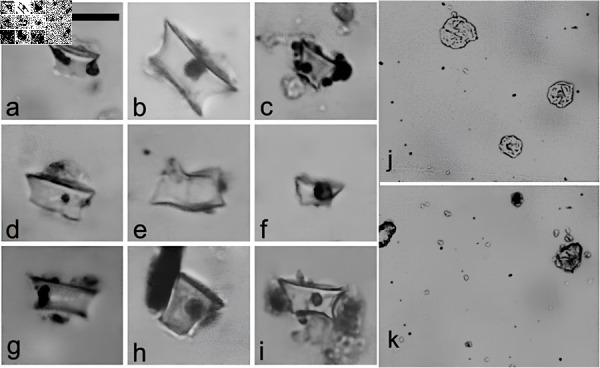
(a–i) *Zea mays* wavy/ruﬄe top rondel phytoliths (scale 20 µm) in LM-FM, (j–k) heat-altered pollen of the same species: residues adhered to the internal surface of the sample No. 3: LM-FM-S1, BIV, P2 in Fig 2, which also corresponds to No. 42 and 43 in Table 1.

In the ceramic sample No. 3: LM-FM-S1, BIV, P2 in Fig 2, which also corresponds to No. 42 and 43 in Table 1, dated at 730 ± 50 years BP, *Saccharomyces yeast* embedded within the vessel wall was identified [5]. Other samples from the same vessel underwent various physical-chemical and taxonomic analyses of microfossils, which revealed the exclusive presence of fatty acids from seeds and berries (Fig 2). Additionally, residues adhered to the internal surface of the vessel were examined, and silico-phytolitic structures similar to those of *Zea mayz* marlo and spike, along with heat-altered pollen of the same species (Fig 3), were identified, alongside other indeterminate plants [31,41].

### Archaeological implications of *S. eubayanus* in America and Europe

The identification of *S. eubayanus* broadened the spectrum of species involved in the domestication processes of nature, including microbes, and raised the hypothesis of an early exchange of products and technologies between America and Europe [2]. In the first case, the importance of domesticating micro and macro species has had significant implications for humanity’s transition from nomadic to sedentary lifestyles. It has expanded the human demography and broadened and complicated both technological and social aspects [42]. However, most domestication studies tend to focus on only a few animal and plant species. Regarding the second implication, historical studies conducted over half a century ago estimated that lager beer production emerged in Bavaria during the $15^{th}$ century [43]. This development was preceded by lighter or blonde beers, which had evolved in Europe since the Middle Ages, particularly Ale-type beers produced using *Saccharomyces cerevisiae*, a yeast also utilized in wine and bread production [43]. However, recent genetic research by Libkind et al. (2011) [2] suggests a later chronology, placing the development of lager strains in the $16^{th}$ century. This aligns with the possibility of an early contribution from the Americas during the period of European conquest. These microbiological findings have sparked debates about the feasibility of such early hybridization with strains of South American origin, challenging traditionally recognized areas of origin [8,33]. As a result, researchers sought alternative explanations, including contributions from other regions on the American continent. Recent records of *S. eubayanus* in trees of the genus Fagus and Acer in Wisconsin, USA, as well as in different species of oaks on the Tibetan plateau, have expanded the geographic distribution of the species’ natural habitat [33,44]. All of these locations were considered potential candidates for hosting cryotolerant strains, a crucial selective factor in the hybridization process of *S. pastorianum* (as discussed in [26]. The initial response was to downplay the hypothesis of a South American contribution proposed by Libkind and collaborators [2]. Instead, researchers considered closer regions, particularly the northern hemisphere, and favored an Asian origin due to historical commercial connections between Europe and the East during the early presence of lager beer. However, studies of nuclear genes (BRE5 and EGT2) and mitochondrial gene (COX2) confirmed the significant contribution of the Patagonian strain of *S. eubayanus*, outweighing the alternatives from the Northern hemisphere of America and Asia [26]. Furthermore, the innovative genetic studies carried out by researchers from the Austral University of Chile confirmed that most of the wild *S. eubayanus* strains collected worldwide belong to the Patagonian group [28].

### The Patagonian connection

The Patagonian hypothesis has a significant ethnohistoric information in its favor, along with novel archaeological evidence [5–7]. Firstly, it is incorrect to consider that commercial circulation between the North Patagonian Andean Forest and Europe was limited to the end of the $19^{th}$ century, as this argument is used to undermine the South American hypothesis. The North Patagonian Andean Forest is an extensive area spanning both sides of the Andes Mountain range, with a long sequence of human occupation dating back to around 14 thousand years BP on the eastern slope and 11,400 years BP in the eastern sector.

Among the earliest dated interactions with forest plant resources during the Pleistocene-Holocene transition, more than 70 plant species were collected, including medicinal plants and others not directly linked to food, such as several species of the genus *Nothofagus sp.* [45]. The use of the North Patagonian Andean Forest environments and La Araucanía in general presents important evidence of human activity, suggesting a specialized way of life in the exploitation of forest resources with a significant component of plant gathering [17,46] and the experimentation of cultigens around the second millennium BP, including the presence of domestic species like *Zea mays* around 1,000 years BP [23,47–49] as well as camelids, widely described in historical sources [48,50]. These pieces of evidence in the western Andean sector, or South Central Area of Chile, have led to the characterization of the southernmost formative societies of the American continent [50], regardless of their Andean roots or lack thereof (see synthesis in Adán et al. 2016 [48]).

### Early European exploration

It is important to clarify that the Andean Forest and lakes were among the earliest areas explored in the interior of continental Argentine Patagonia by the Hispanic conquistadors. They moved from the Pacific coast and occupied strategic enclaves on the eastern mountain range, establishing forts such as Imperial, Concepción, and Valdivia to the south of the Bío Bío river. However, these forts were repeatedly destroyed by the Reche-Mapuche indigenous people. Despite this, they were reoccupied on different occasions by European conquerors, including a two-year Dutch occupation before being displaced again by Hispanics [51].

The Hispanic chronicles of the $16^{th}$ century described aggregation ceremonies across extensive territories that included the eastern Andean slope. These ceremonies involved large amounts of food and fermented drinks, often driven by the search for sources of gold and silver.

It is essential to highlight that the “Wallmapu” or Mapuche ancestral territory, which occupied both slopes of the mountain range, has a long history identified through the archaeological record [52]. The first pottery occupations of the North Patagonian Andean forest and lake area date back around 2,000 years BP [10,48]. This territory is the natural habitat of the species of Nothofagus and *Araucaria araucana*, hosts of the cryotolerant yeast species that are the focus of the present work (Fig 1). Recent archaeological evidence suggests [7] that pots containing potentially fermentative residues of *Araucaria araucana* pine nut seeds from the Cordillera forests were transported more than 600 km eastward, reaching sectors of the upper Río Negro basin approximately 1,000 years ago. This indicates that five centuries prior to the arrival of Europeans, fermented drinks made from resources of the Cordilleran forests were highly valued by various native societies. These drinks traveled great distances, likely as prestige and ceremonial goods, as well as food exchanged between producer societies and hunter-gatherer-potter eastern neighbors.

### Phylogenetic and phylogeographic results

**Fig 4:**
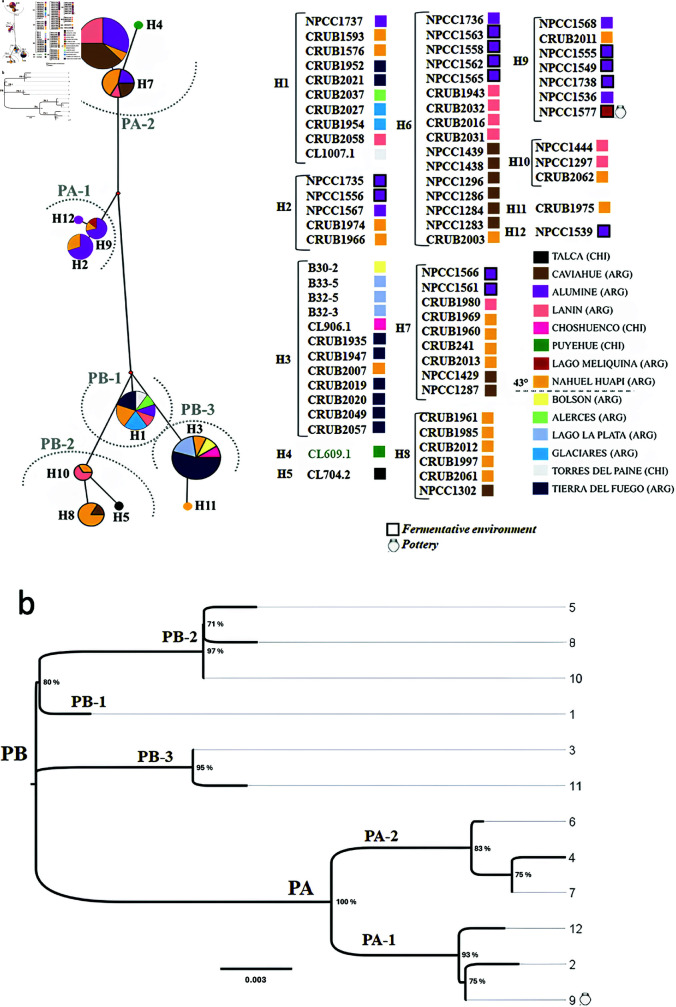
Phylogeografic and phylogenetic analysis of *S. eubayanus strains.* (a) Median Joining (MJ) network net reconstructed using the *IntFR* partial sequences. Circles represent individual haplotypes, and the circle size is correlated with the number of strains of each haplotype. The legend of the strains belonging to each haplotype is in the upper right box. The colors of each haplotype indicate the sampling area where they were isolated. Boxes outlined with a black border represent strains isolated from fermentative environments. (b) Neighbor-Joining (NJ) phylogeny of 72 strains based on (441pb positions in the final dataset) *IntFR* region. The tree is drawn to scale, with branch lengths in the same units as those of the evolutionary distances used to infer the phylogenetic tree. The evolutionary distances were computed using the Tamura-Nei model [36] method and expressed as the number of base substitutions per site. The two clades are marked by letters PA and PB. NCBI accession numbers are included in the Data availability statement.

Both the phylogeographic (Fig 4a) and phylogenetic (Fig 4b) analyses, obtained from the analysis of the *IntFR* region, separate the two genomic populations and the five subpopulations described for the species *S. eubayanus* [28,29,53]. The strain isolated from the vessels belongs to the PA-1 subpopulation and has a different haplotype from all the other strains analyzed. This indicates that there was no contamination with current strains, at least when considering those used in this analysis (Fig 4). All these strains were isolated from Alumine in apple chichas or feral apple trees (with the exception of the strain CRUB2011 isolated from natural environment in Nahuel Huapi).

Regarding the geographic data, the PA-1 population has only been described in the regions that make up the Nahuel Huapi Park and its surroundings. These data are correlated with the strain isolated in vessels, since its sampling site (Lake Meliquina) falls between the two geographical locations of the strains CRUB1974 (Ñirihuau, Argentina)and CRUB1966 (Piedras Blancas, Argentina) with which it presents the greatest genomic similarity (Fig 4a).

On the other hand, the PA population is the one that presents the least similarity with the isolated populations from the rest of the world (United States, Tibet, and New Zealand), and it could be said that its distribution is completely restricted to Patagonia [54]. This fact, added to the observation that it is systematically more basal than the PB population [53], reinforces the hypothesis that the strains of the PA population were those involved in the fermentative processes 920-750 years ago.

Regarding the fermentative capacity of these subpopulations, previous studies in malt extract wort have reported a lower attenuation capacity for strains of the PA-1 genomic subpopulation compared to other subpopulations. However, this difference is less evident when evaluating CO_2_ production and fermentation rates, suggesting that these strains are indeed fermentative [29]. The authors attribute the lower attenuation to the inability of PA-1 strains to metabolize maltotriose. Nevertheless, since maltotriose is not a carbon source typically present in corn, these strains could still perform effectively as fermenters when utilizing corn-based substrates, such as those found in the pottery samples from Lake Meliquina (Figs 2 and 3).

In a recent study, we investigated the fermentative capacity of these subpopulations and discovered that they exhibit higher ethanol tolerance compared to other subpopulations. Moreover, strains belonging to this subpopulation demonstrated the highest implantation percentage in natural fermentations of apple must [30].

The discovery of an identical haplotype in the strain isolated from vessels and others isolated from fermentative environments strengthens the hypothesis that this strain may have played a role in fermentations dating between the years 920–750. However, this finding also motivates us to investigate other genomic regions in search of variations that could be used to demonstrate the evolutionary divergence between this strain and contemporary ones. One potential avenue for exploration is the utilization of the *IntFR *region. Future research should consider conducting whole genome sequencing analyses for this strain to further support our theories.

On the other hand, the absence of internal surface alterations, such as acid etching or pitting, is linked to the typological groups of pottery found at the sites, particularly in the analyzed samples. These vessels appear to be more closely associated with the containment and serving of liquids rather than the production of fermented beverages. This suggests limited access to fermentative drinks or their containment and serving in a small number of vessels, such as the pot analyzed in LM-FS, S1. These ceramics, from hunter-gatherer societies, contain traces of food products but are considered a minority. In that sense, fermentation could be had used by these societies as a potential method for utilizing seasonally abundant plant resources [23].

## Discussion

The identification of cryotolerant fermentative yeasts through phylogeographic and phylogenetic analyses conducted on yeast remnants found within the walls of ceramic vessels, dating between 920 and 750 years BP at Lago Meliquina, coincides with indirect records indicating the fermentation practices involving grass and fruit at Mocha Island and Angostura 1. Therefore, the identification and context of the *S. eubayanus* strain in Lago Meliquina constitute another proxy that reinforces the hypothesis about the southernmost pre-Hispanic development area of the continent for the production of fermented beverages, between $-38^{\circ }$ and $-40^{\circ }$ south latitude.

Together with *S. eubayanus*, silico-phytolithic residues and heat-altered starches of *Zea mays* [23,31] were identified, similar to findings on Mocha Island [6]. As demonstrated by the residues previously identified in the ceramics of Lago Meliquina (Table 1, Figs 2 and 3). Additionally, potentially fermentative seeds and fruit fatty acids were also absorbed in the walls of some vessel [31]. Based on these multiple evidences, the presence of *S. eubayanus* yeast is postulated as a by-product of at least the consumption and/or serving of fermented beverages among ceramic vessels at the site.

This finding confirms that *S. eubayanus was* used for the fermentation of various seeds and/or native fruits in our study area before the incorporation of the commercial baker’s yeasts registered by Rodríguez et al. (2014) [26], and that traditional fermentations of apple must [30] continue to dominate today.

The isolation of *S. eubayanus* from pre-Hispanic ceramic samples at Lake Meliquina (Table 1), along with its identification as a primitive strain (PA1) through phylogeographic and phylogenetic analyses (Fig 4), and its direct association with silico-phytolithic residues and heat-altered corn remains (Figs 2 and 3), support the hypothesis that this yeast was part of the residual material absorbed by the walls of ceramic containers prior to European-Indigenous contact. This provides evidence of the potential management or domestication of *S. eubayanus* for its role in the fermentation of native seeds and berries, even before its later hybridization for beer production in Europe. As a result, we hypothesize that this yeast may have been introduced into Europe from South America during the early circulation of people and various material objects in the first half of the $16^{th}$ century. According to the findings of Libkind et al. (2011) [2], genetic adaptations likely enabled *S. eubayanus*—which exists as a predominantly pure lineage in natural conditions in Patagonia—and later *S. pastorianus*, which shares 99.5% genetic similarity, to thrive in cold fermentation conditions, a key feature of lager brewing. The strain could have circulated as contamination of containers that contained fermented native drinks, but these same drinks could also have been part of the rations of the conquerors for consumption during the sea voyage or in their place of origin, among other exotic goods that circulated from America to Europe.

## Conclusion

This research confirms, through genetic studies on pottery residues, the pre-Hispanic use of the cryotolerant fermentative yeast *Saccharomyces eubayanus* in the fermentation of native fruits and seeds. Additionally, phylogenetic and phylogeographic evidence highlights the involvement of ancient strains of this yeast from the southern cone of America in the hybridization processes in Europe during the mid-sixteenth century. These processes, whether intentional or not, contributed to the development of blonde or lager beers.

## Supporting information

S1 Table National Center for Biotechnology Information (NCBI) accession numbers. (XLSX)
